# A Painful Subscapular Mass

**Published:** 2020-04-26

**Authors:** Alexandra Naides, Jerette Schultz, Ashley Ignatiuk

**Affiliations:** Rutgers New Jersey Medical School, Newark

**Keywords:** elastofibroma dorsi, subscapular, lipoma, mass, back

## DESCRIPTION

A 49-year-old woman presented with a left back mass in the subscapular region that began to cause pain with shoulder movement. Ultrasound scan was obtained to evaluate the lesion and showed a 6.9 × 6.3-cm mass between the left scapula and the posterior chest wall. It was 1-cm thick, discoid, well-circumscribed, and heterogeneous in nature and diagnosed by the radiologist as most likely elastofibroma dorsi based on the location. The patient was taken to the operating room and a fatty soft-tissue mass was removed from beneath the serratus anterior muscle. The final pathology report revealed the mass to be a lipoma.

## QUESTIONS

1. What is elastofibroma dorsi?
2. How is elastofibroma dorsi diagnosed and what is the differential diagnosis?
3. What are the surgical indications for subscapular masses and the potential complications?
4. What is the surgical approach to subscapular masses?

## DISCUSSION

Elastofibroma dorsi is a rare, benign, soft-tissue tumor that usually develops between the tip of the scapula and the posterior chest wall. Traditionally, elastofibroma dorsi is located at the inferior border of the scapula between the serratus anterior and the chest wall anteriorly and the latissimus dorsi muscle posteriorly. However, there are a few case reports in the literature of elastofibromas occurring in other regions of the body, including the greater trochanter, ischial tuberosity, olecranon, axilla, elbow, hand, foot, tricuspid valve, stomach, rectum, eye, and inguinal region.^1^ Elastofibroma dorsi tends to develop in women older than 50 years. Most case series show a female predominance, with one study finding the female-to-male ratio to be as much as 5:1, with a mean age of diagnosis being 65 years.^2^ Histologically, the tumor is characterized by elastic fibers in various stages of maturation surrounded by collagen and fatty connective tissue.^1^ The etiology of this tumor is not well known, but there are few predominant theories in the literature.^3^ The first theory suggests that overuse of the scapula produces mechanical friction that stimulates fibroblasts to produce elastic tissue.^3^ The second possible etiology postulates that elastofibromas do not form from a degenerative process alone but rather there is a component of abnormal elastogenesis secondary to vascular insufficiency involved as well.^3^ The third theory suggests that enzymatic instability and a possible hereditary predisposition contribute to the formation of an elastofibroma.^3^

Many patients will present with a noticeable mass in their subscapular region.^4^ The average size of these tumors in one case series ranged from 3 to 13 cm.^4^ Similarly, another case series found the tumor size to be 45 to 110 mm in diameter.^5^ The relatively large size of these tumors may be due to the fact that some patients do not seek medical attention until they become large enough to cause symptoms or undesired cosmesis.^4^ The most common presenting symptoms are pain and “clunking” or “snapping” of the scapula with abduction and adduction of the shoulder.^4^^-^6 Swelling of the shoulder and dyspnea were less common, but they have also been reported as associated presenting symptoms.^4^ Many imaging modalities have been used to assess elastofibroma dorsi tumors, including ultrasonography, computed tomography (CT), magnetic resonance imaging (MRI), and positron emission tomography–computed tomography. On occasion, these tumors are diagnosed incidentally when the patient undergoes imaging for another reason. On the ultrasound scan, elastofibroma dorsi generally appears in the characteristic location as a mass with alternating areas of hyperechogenic and hypoechogenic lines that can parallel the chest wall.^7^ However, CT and MRI are the more commonly used imaging modalities to diagnose soft-tissue tumors of the chest wall. On the CT scan, elastofibroma is typically a poorly defined heterogeneous soft-tissue mass containing linear streaks of fat and attenuation similar to skeletal muscle.^2^ On the magnetic resonance image, soft-tissue appearance is similar to that of skeletal muscle on both T1- and T2-weighted images with strands of soft tissue having intensity resembling fat.^2^ The differential diagnoses for soft-tissue tumors of this type include elastofibroma dorsi, desmoid tumors, sarcomas, lipomas, fibromas, neurofibromas, and aggressive fibromatosis.^2^^,^^4^ There are case reports in the literature of lipomas occurring in the subscapular region that can present with symptoms such as winged scapula and radicular neuropathy.^8^ However, the typical position of the tumor occupying the subscapular space anterior to the rib cage, age of the patient, and female gender can help narrow the diagnosis and are suggestive of an elastofibroma.^4^ Following the administration of a contrast agent, tumors that met the radiologic criteria for elastofibroma dorsi can show enhancement not unlike malignant tumors.^4^ Therefore, biopsy or excision is the only way to confirm tissue diagnosis and truly rule out malignancy.

Because these types of subscapular masses are relativity rare, management is based on a limited number of small case series. There are no clearly defined treatment guidelines. The predominant symptoms that prompt patients to seek medical attention are pain, snapping of the scapula, and swelling.^4^^-^6 The main indication for surgical resection in subscapular tumors is the severity of the patient's symptoms. However, given the extensive differential diagnosis, there are several areas to consider when determining a treatment plan. First, the benefits of surgical resection should be weighed against the risk of complications. Multiple case series that examined surgical resection of elastofibroma dorsi tumors describe hematoma and seroma as the most frequent complications of surgical resection. The incidence of postoperative hematoma ranged from 10.5% to 43% depending upon the case series.^5^^,^^6^ The postoperative seroma rate was reported anywhere from 26.3% to 85.7% depending on the cohort.^4^^,^^6^ When controlled for age, hypertension, preoperative symptoms, and intraoperative bleeding, there was no statistically significant difference between the patients who developed hematomas and those who did not.^5^ However, tumor size and length of postoperative drainage were significantly different when comparing the 2 groups.^5^ In some instances, these complications were managed conservatively whereas others required surgical evacuation or fine-needle aspiration.^5^^,^^6^ Second, the possibility of a malignant tumor on tissue diagnosis or the possibility of malignant transformation in benign lesions should be considered. There are no reports of malignant transformation of elastofibroma dorsi in the literature. Therefore, given the benign nature of this tumor, asymptomatic patients can be treated with conservative management once the diagnosis has been confirmed. Local recurrence of elastofibroma is also rare, with only a few case reports in the literature.^4^ At a median duration of active follow-up of 5 months (0-121 months), patients managed nonoperatively did not show worsening symptoms, suggesting long-term follow-up is not necessary.^6^

**Figure 1:**
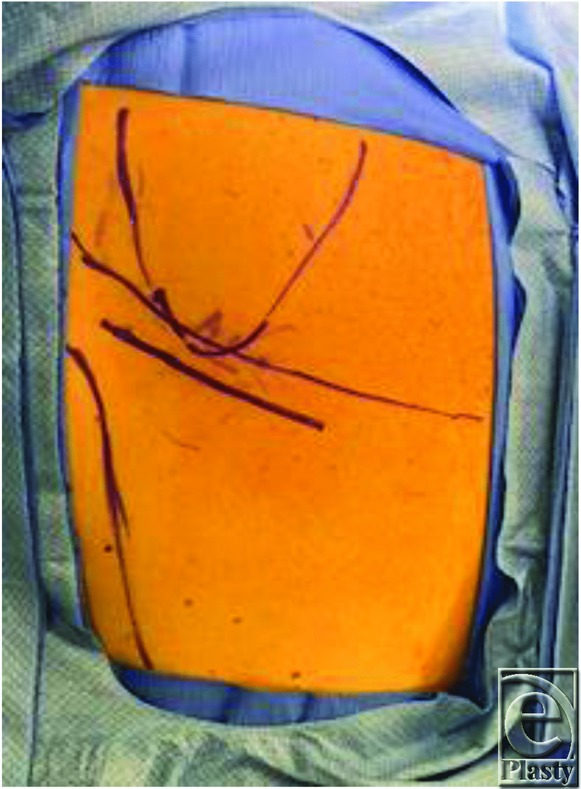
Preoperative markings outlining the scapula, latissimus dorsi muscle and planned oblique incision approximately 2cm below the angle of the scapula.

**Figure 2:**
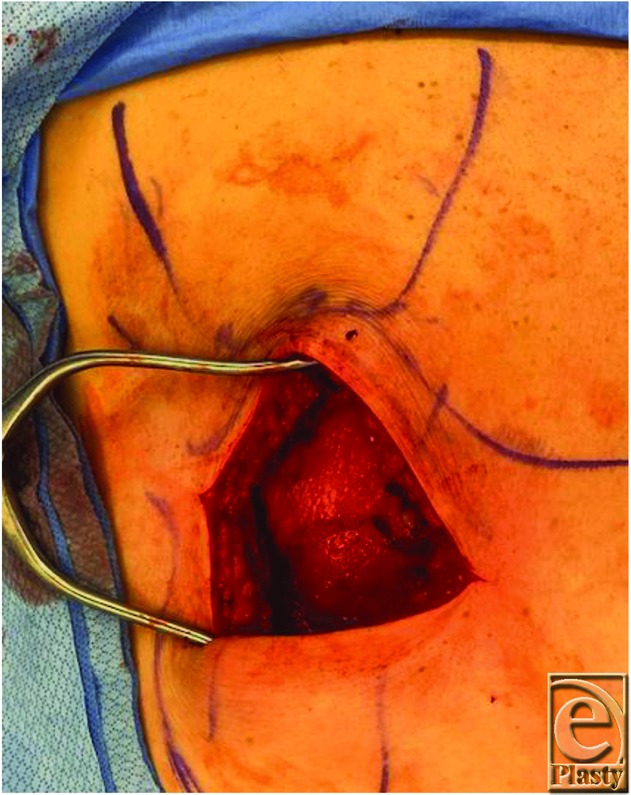
During dissection, the latissimus dorsi is split in line with its muscle fibers.

**Figure 3:**
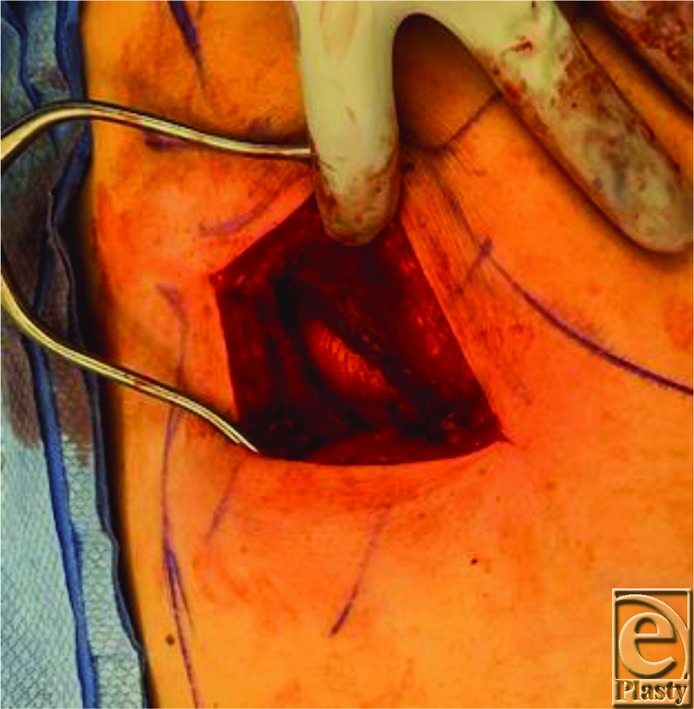
The mass is visualized under the serratus anterior after splitting it in line with its muscle fibers.

**Figure 4:**
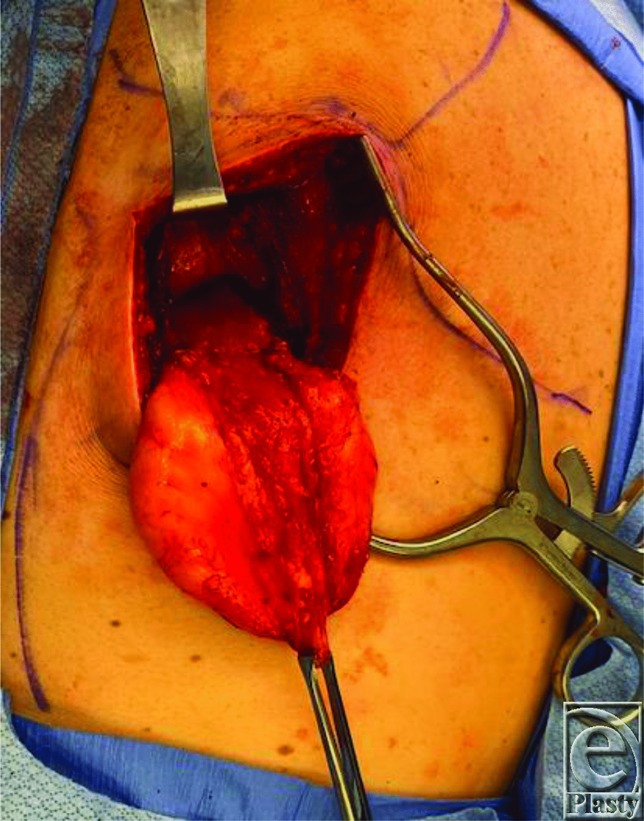
The mass delivered outside of the wound after being dissected off the posterior chest wall.

**Figure 5:**
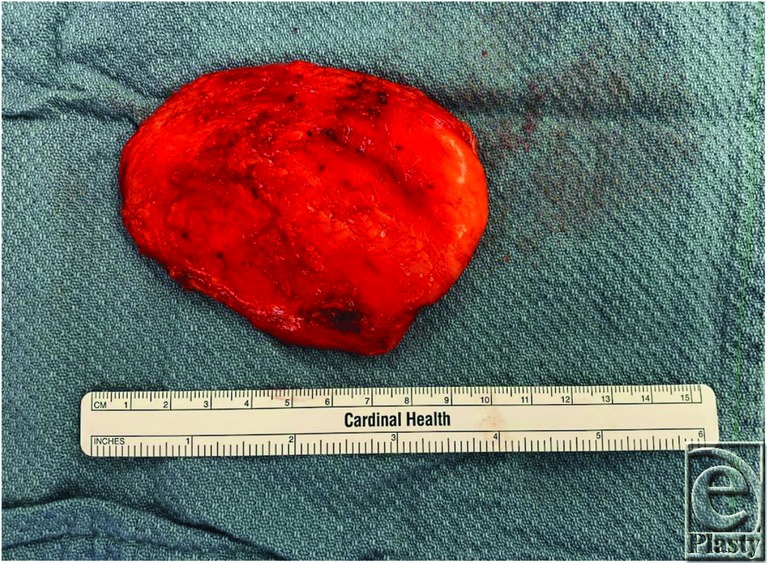
The mass after complete excision, measuring about 10cm in greatest dimension.

It is helpful to obtain imaging for subscapular masses to aid in diagnosis and for surgical planning. Ultrasound scan showed the position of the mass in this case to overly the posterior chest wall caudal to the inferior pole of the scapula and underneath the serratus anterior muscle, as is the typical location for an elastofibroma dorsi. During surgery, the patient is placed in prone position on the operating room table for ease of access. For patients who cannot tolerate being prone for anesthesia reasons, excision can also be done in the lateral position. It is helpful to mark the inferior border of the scapula as well as the latissimus dorsi muscle preoperatively. The skin incision is designed to be about 10 cm oriented obliquely in line with fibers of the latissimus dorsi muscle, approximately 2 cm beneath the angle of the scapula (Fig 1). Dissection is carried down through the skin and subcutaneous tissue, and the latissimus dorsi muscle is identified and split in line with its fibers using electrocautery (Fig 2). Small motor branches from the thoracodorsal nerve should be identified and spared when possible. The serratus anterior muscle is then identified beneath the latissimus dorsi and is also split at its inferior edge in line with its fibers (Fig 3). Palpation of the mass at this point can help direct where the splitting of the serratus muscle should occur. The mass is then identified and dissected circumferentially underneath the scapula and off the chest wall (Fig 4). The mass in this case was found to be fatty in nature and encapsulated (Fig 5); however, elastofibroma dorsi can be somewhat adherent to the periosteum of the posterior ribs and dissection should be meticulous with careful hemostasis. After the mass is resected, the muscle fibers of the serratus anterior and latissimus dorsi are loosely re-approximated with absorbable suture. Because of the dead space created and a high rate of hematoma and seroma, 1 to 2 drains should be left in place for approximately 1 week or until the output has decreased to less than 30 mL per day. In this case, one drain was left in the large dead space under the scapula and another drain was left under the latissimus dorsi.

Although painful subscapular masses between the posterior chest wall and tip of the scapula can be pathognomonic of elastofibroma dorsi, more common soft-tissue masses such as lipoma can also present in this anatomic area. No matter the final tissue diagnosis, surgical resection is indicated to relieve patient symptoms.
